# Spatiotemporal distribution characteristics and disease burden of human brucellosis in Inner Mongolia Autonomous Region, 2014–2023

**DOI:** 10.3389/fpubh.2026.1896637

**Published:** 2026-09-16

**Authors:** Qi Zhang, Junyu He, Zhiruo Zhu, Benjian Lan, Hua Shi, Zhongbing Zhang, Na Ta, Qiulan Chen, Yuanyong Xu, Wenyi Zhang

**Affiliations:** 1 Department of Epidemiology, School of Public Health, China Medical University, Shenyang, China; 2 Chinese PLA Center for Disease Control and Prevention, Beijing, China; 3 Ocean College, Zhejiang University, Zhoushan, China; 4 Inner Mongolia Autonomous Region Center for Disease Control and Prevention, Hohhot, China; 5 National Key Laboratory of Intelligent Tracking and Forecasting for Infectious Disease, Chinese Center for Disease Control and Prevention, Beijing, China

**Keywords:** brucellosis, disease burden, Inner Mongolia, Joinpoint, spatiotemporal distribution

## Abstract

**Background:**

This study aimed to characterize the spatiotemporal dynamics and disease burden of human brucellosis in Inner Mongolia from 2014 to 2023, providing a robust scientific foundation for tailored prevention and control strategies.

**Methods:**

Descriptive epidemiological analysis and Joinpoint regression models were employed to evaluate the temporal trends and multidimensional distribution of the epidemic. Spatial autocorrelation and spatiotemporal scan statistics were utilized to identify spatiotemporal clusters at the county level. Furthermore, the disease burden was quantified using years lived with disability (*YLDs*) and estimated indirect economic losses.

**Results:**

The overall incidence of brucellosis in Inner Mongolia exhibited a significant upward trend from 2014 to 2023 (AAPC = 10.42%, *p* < 0.001). Joinpoint analysis identified a pivotal inflection point: incidence rates declined between 2014 and 2016, followed by a sharp resurgence from 2016 to 2023 (APC = 19.75%, *p* < 0.05). Regional disparities were substantial. Eastern leagues exhibited marked epidemiological heterogeneity, whereas western leagues experienced considerable increases in incidence, with the exception of Alxa League. Male farmers and herdsmen constituted the most vulnerable population affected by the epidemic. Significant spatiotemporal clustering and a pronounced seasonal peak in spring–summer (March–July) were identified, coinciding with the regional livestock breeding season. High-incidence clusters were predominantly distributed across central and eastern traditional pastoral regions, with core epidemic clusters persisting for approximately five consecutive years. Over the decade, the cumulative disease burden reached 4,033.61 person-years in *YLDs*, resulting in an estimated CNY 204.88 million (approximately USD 28.95 million) in indirect economic losses. This burden exhibited marked disparities across time, geography, and age groups.

**Conclusion:**

Brucellosis prevention and control in Inner Mongolia should prioritize regions with moderate economic development. Veterinary and public health facilities should be strengthened to adapt to the ongoing transformation of animal husbandry. Targeted efforts should focus on expanding livestock vaccination coverage and optimizing protection protocols for occupationally exposed populations. Implementation of interventions in advance of the spring and summer epidemic peaks is vital to effectively contain disease transmission.

## Highlights

- Incidence fell 2014-2016, then sharply surged until 2023 (APC=19.75%).
- Male farmers and herdsmen were the population most affected by the epidemic.
- Core spatial clusters persisted in central-eastern and southern pastoral areas for ~5 years.
- A decade-long burden reached 4,033.61 YLDs and 204.88 million CNY in losses.

## 1 Introduction

Brucellosis, a debilitating zoonotic disease caused by bacteria of the genus Brucella, is classified by the World Health Organization (WHO) as a neglected yet priority-controlled public health issue. It poses a persistent and severe challenge to global public health security and the livestock economy (1). Human transmission typically occurs through direct contact with infected animal tissues, the ingestion of unpasteurized dairy products, or the inhalation of contaminated aerosols. Clinical manifestations are predominantly characterized by undulant fever, profuse diaphoresis, and osteoarticular pain. Progression to the chronic stage in some patients can lead to significant impairment of labor capacity and quality of life (2). Although official WHO data indicate over 500,000 new cases annually worldwide, the actual disease burden far exceeds monitored levels due to diagnostic delays and systemic under-reporting (3).

Research has demonstrated that brucellosis imposes a profound dual burden on both public health and the economic stability in endemic regions (4, 5). A 2015 WHO global assessment of 31 foodborne diseases revealed that the global burden of brucellosis reached as high as 124,884 Disability-adjusted Life Years (*DALYs*) (6). In Kazakhstan, annual economic losses attributed to brucellosis are approximately USD 21 million (7), Similarly, the average total cost per patient in Iran reaches USD 1,060, with direct medical costs accounting for 73.6% of the total expenditure (8); In China, the average annual disease burden from 2004 to 2017 was estimated at 5,175 *DALYs*, with an indirect economic burden of USD 41.02 million. Furthermore, the median direct cost per brucellosis episode was USD 688.65, a figure that exceeds the national per capita health expenditure and exhibits a continuous upward trend (9, 10).

In China, brucellosis has spread across 31 provinces, exhibiting significant geographical heterogeneity (11), As China’s critical livestock hub, the Inner Mongolia Autonomous Region has consistently reported the highest incidence nationwide, with an average annual incidence rate of 52.59 per 100,000—substantially exceeding the national average (12, 13). Although previous spatiotemporal dynamic studies have confirmed prominent seasonal and spatial clustering within this region, quantitative research identifying definitive turning points in epidemic trends over the past decade remains scarce. In particular, the lack of clarity regarding key inflection points in incidence rates has hindered the scientific evaluation of the effectiveness of prevention and control strategies (14, 15). To fill this research gap, we analyzed population-based surveillance data on human brucellosis in Inner Mongolia spanning 2014 to 2023, characterizing its temporal trends, spatiotemporal clustering patterns, and associated disease burden. By identifying high-risk regions, key trend turning points and population-specific disease burden over the decade, this study generates evidence to inform targeted intervention strategies, with the ultimate aim of mitigating the dual impacts of brucellosis on public health security and socioeconomic development in Inner Mongolia.

## 2 Materials and methods

### 2.1 Study area

The Inner Mongolia Autonomous Region is situated along the northern frontier of China (37°24’-53°23’ N, 97°12’-126°04’E). It covers a total area of 1.183 million km^2^, accounting for 12.3% of China’s total landmass and ranking third among all provincial-level administrative divisions. By the end of 2025, the region reported a permanent resident population of 23.74 million, comprising 16.97 million urban residents and 6.77 million rural residents. Administratively, the region exercises jurisdiction over 9 prefecture-level cities and 3 leagues, encompassing a total of 103 banners, counties, and districts (16). Inner Mongolia was selected as the study area because it has long been a recognized endemic region for brucellosis and has consistently reported the highest number of human cases nationally. Despite the launch of an animal brucellosis control program in 2009, the epidemic has since resurged, and the region is now considered one of the areas with the most severe animal brucellosis burden in China. A national meta-analysis of bovine brucellosis in China (2014–2024) reported that Inner Mongolia had the second-highest cattle seroprevalence among all provinces, at approximately 3% (17). The region has also been classified as a Type I brucellosis severe epidemic region for sheep and goats, reflecting its position as the province with the largest sheep population in China (18), and by the end of 2023 its livestock inventory included approximately 9.48 million cattle and 61.81 million sheep and goats (19). Census data from 2020 show that 32.5% of the region’s population resided in rural areas, where livelihoods are mainly based on farming and animal husbandry (20). This proportion can serve as an approximate indicator of the share of residents with regular occupational or household-level livestock contact.

### 2.2 Data sources

Case data for the Inner Mongolia Autonomous Region from 2014 to 2023 were obtained from the local Center for Disease Control and Prevention (CDC). All cases were diagnosed in strict accordance with the criteria and principles defined in the “Diagnosis for Brucellosis” (WS 269–2019), a health industry standard of the People’s Republic of China (21). The data were aggregated to a monthly level for each district and county. To ensure confidentiality, all patient records were anonymized, ensuring that no personally identifying information was involved in this study (Figure 1).

**Figure 1:**
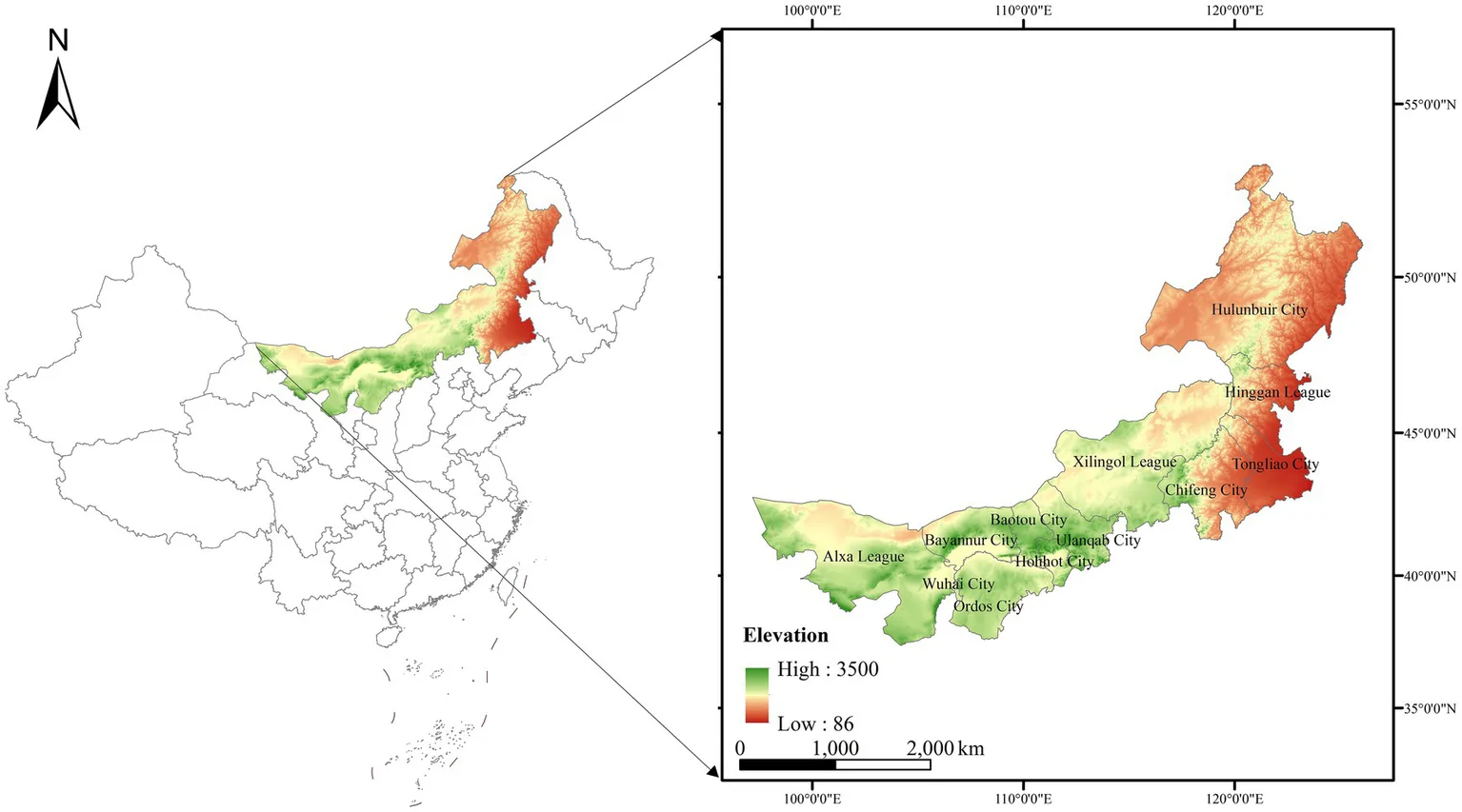
Study area location in Inner Mongolia Autonomous Region. Map data source: National Platform for Common Geospatial Information Services. (https://cloudcenter.tianditu.gov.cn/administrativeDivision/).

### 2.3 Joinpoint regression model

Segmented log-linear models were constructed using the Joinpoint Regression Program 5.3.0 to analyze the temporal trends in reported brucellosis incidence in the Inner Mongolia Autonomous Region. The annual percentage change (APC) for each segment was calculated as:

APC=100×(exp(β)−1)

Where β is the regression coefficient of that segment from the fitted segmented log-linear model. The average annual percentage change (AAPC) across the study period was calculated as a weighted average of the segment-specific slopes, using the length of each segment within the specified interval as the weight:

$$\text{AAPC}=100\times (\exp(\Sigma w_{i}\beta_{i}/\Sigma w_{i})-1)$$

Where βᵢ is the regression coefficient of the i-th segment and wᵢ is the number of years that segment falls within the study period. Ninety-five percent confidence intervals (CIs) for both APC and AAPC were derived from the model output. A 95% CI entirely above 0 was considered to indicate an increasing trend, entirely below 0 a decreasing trend, and a CI spanning 0 a stable trend (22, 23). An APC or AAPC > 0 indicates an upward trend, while a value < 0 denotes a downward trend. A scenario where APC equals AAPC represents a monotonic trend (either purely increasing or decreasing) (24, 25).

### 2.4 Spatio-temporal distribution characteristics

In the Global Moran Index, values close to 0 indicate absence of spatial autocorrelation, and the closer the variation is to –1 and +1, it indicates negative or positive correlation (26). To further delineate localized risk hot spots, retrospective spatiotemporal scan statistics based on a discrete Poisson model were deployed at the county level using SaTScan. The maximum spatial cluster size was restricted to 10% of the population at risk, with statistical significance evaluated via 999 Monte Carlo simulations at a threshold *p* value less than 0.05. Candidate clusters were subsequently ranked by their log-likelihood ratios (LLRs) to define the most likely cluster (MLC) and secondary clusters (21).

### 2.5 Disease burden

Disability-Adjusted Life Years (*DALYs*) primarily consist of two components: Years of Life Lost (*YLL*) due to premature mortality and Years Lived with Disability (*YLD*). The relationship is defined as.

DALY=YLL+YLD

Where YLL represents healthy years of life lost due to premature death, and *YLD* represents healthy years lost due to disability. Given the extremely low mortality rate of human brucellosis, *DALYs* in this study were assumed to be approximately equal to YLD (*DALY*≈*YLD)*, the specific formula for *YLD* is as follows:

$$\mathrm{YLD}=I\times D_{w}\times L$$

In this formula, *YLD* denotes the years lived with disability caused by a specific disease within a time period *t*. *I* represents the number of new human brucellosis cases during the study period; *D_w_* is the disability weight (ranging from 0 to 1), assigned as 0.15 (27) for human brucellosis in this study. *L* refers to the average duration of the case from onset to recovery or death (in years). According to official data from the WHO, the average duration of brucellosis is approximately 75 days (28).

Indirect economic burden refers to the economic losses resulting from reduced or lost productivity due to illness, disability, or premature death. This study calculates the indirect economic burden of human brucellosis based on the Human Capital Approach, utilizing international standard productivity weights for different age groups: 0.15 for 0–14 years, 0.75 for 15–44 years, 0.80 for 45–59 years, and 0.1 for those aged 60 and above (29). The formula is defined as:

$$\begin{array}{l} \text{Indirect Economic Burden}=\mathrm{Per}\;\text{Capita}\;\mathrm{GDP}\\ \times \text{DALYs}\times \text{Productivity Weights} \end{array}$$

### 2.6 Software and statistical significance

Data organization and statistical analysis were performed using Microsoft Excel 2021 and R software (version 4.5.1). Joinpoint regression models were employed to evaluate the temporal trends in human brucellosis incidence across the Inner Mongolia Autonomous Region and its constituent sub-regions. Geographical distribution maps of the cases were generated using ArcGIS 10.8, while spatio-temporal scan analysis was conducted with SaTScan 10.2.4. Statistical significance was defined as *p* < 0.05. Currency conversion (CNY to USD) was based on the 2023 average exchange rate (1 USD = 7.0774 CNY).

## 3 Results

### 3.1 Demographic and Joinpoint regression analyses results

A total of 130,855 cases of human brucellosis were reported between 2014 and 2023. The male-to-female ratio was 2.2:1; and the median age of the patients was 49 years, with the highest concentration of cases occurring in the 50–59 age group. Individuals aged 30–69 accounted for 87.9% of all cases. Farmers and herdsmen constituted the majority of the patients (87.98%) (Table 1). Temporal analysis of the monthly aggregated case data revealed a distinct seasonal pattern, with cases concentrated between March and July, which together accounted for 55.3% of all reported cases over the study period. The incidence peaked in May (12.14% of total cases), followed by June (11.72%), while the lowest monthly proportions were observed in September and October (5.37 and 4.99%, respectively).

**Table 1:** Demographic characteristics of human brucellosis in Inner Mongolia, 2014–2023.

Variable	Total (*N* = 130,855)
Sex	
Male	90,211 (68.94%)
Female	40,644 (31.06%)
Median age (years) (IQR)	49 (40, 57)
Age group (years)	
0–9	1,052 (0.80%)
10–19	1708 (1.31%)
20–29	8,136 (6.22%)
30–39	21,633 (16.53%)
40–49	34,916 (26.68%)
50–59	37,549 (28.70%)
60–69	20,924 (15.99%)
≥70	4,937 (3.77%)
Occupation	
Farmers or and herdsmen	115,130 (87.98%)
Children and students	2,349 (1.80%)
Others	13,376 (10.22%)
Median interval from illness onset to diagnosis (days) (IQR)	15 (7, 31)

Joinpoint regression analysis revealed a single inflection point in the epidemiological trend of human brucellosis in Inner Mongolia from 2014 to 2023. A downward trend was observed from 2014 to 2016 (APC = −16.88, *p* < 0.05), followed by a significant upward trend from 2016 to 2023 (APC = 19.75, *p* < 0.05). Overall, the incidence rate across the region showed an increasing trend during the study period, with an AAPC of 10.42 (*p* < 0.001). In eastern Inner Mongolia, one inflection point was identified, respectively, for Hulunbuir, Chifeng, and the Hinggan League, where the epidemic was characterized by alternating patterns of resurgence and growth. No joinpoints were detected for Tongliao or the Xilingol League. The upward trends were highly consistent across the leagues and cities of western Inner Mongolia. Except for the Alxa League, significant increases in incidence rates (*p* < 0.05) were observed from 2014 to 2023 in Hohhot, Baotou, Ordos, Ulanqab, Bayannur, and Wuhai. Distinct inflection points were noted in Hohhot, Baotou, and Ulanqab, transitioning from an initial period of relative stability or short-term fluctuation to rapid growth. In Ordos and Bayannur, incidence rates exhibited a continuous climb. Overall, the epidemic in the western region showed a significant escalating trend, with AAPC values ranging from 3.25 in Hohhot to 21.43 in Ordos **(**Table 2, Figure 2).

**Table 2:** Joinpoint regression analysis: trends in the incidence rate of human brucellosis in Inner Mongolia, 2014–2023.

Region	AAPC	95% CI(%)		*p*-values
		Lower CI	Upper CI	
Total	10.42	5.48	18.00	<0.001
Eastern Inner Mongolia				
Hulunbuir City	2.69	−1.79	10.18	0.22
Hinggan League	10.50	2.01	20.40	0.01
Tongliao City	19.88	−1.31	44.77	0.06
Chifeng City	5.81	−6.65	29.78	0.29
Xilingol League	0.99	−7.76	10.59	0.82
Western Inner Mongolia				
Hohhot City	3.25	0.12	6.87	0.04
Baotou City	14.00	5.42	27.17	<0.001
Ordos City	21.43	15.05	28.58	<0.001
Ulanqab City	13.61	5.63	22.73	<0.001
Bayannur City	19.85	12.80	27.02	<0.001
Wuhai City	17.53	8.17	30.74	<0.001
Alxa League	6.52	−1.00	14.18	0.09

**Figure 2:**
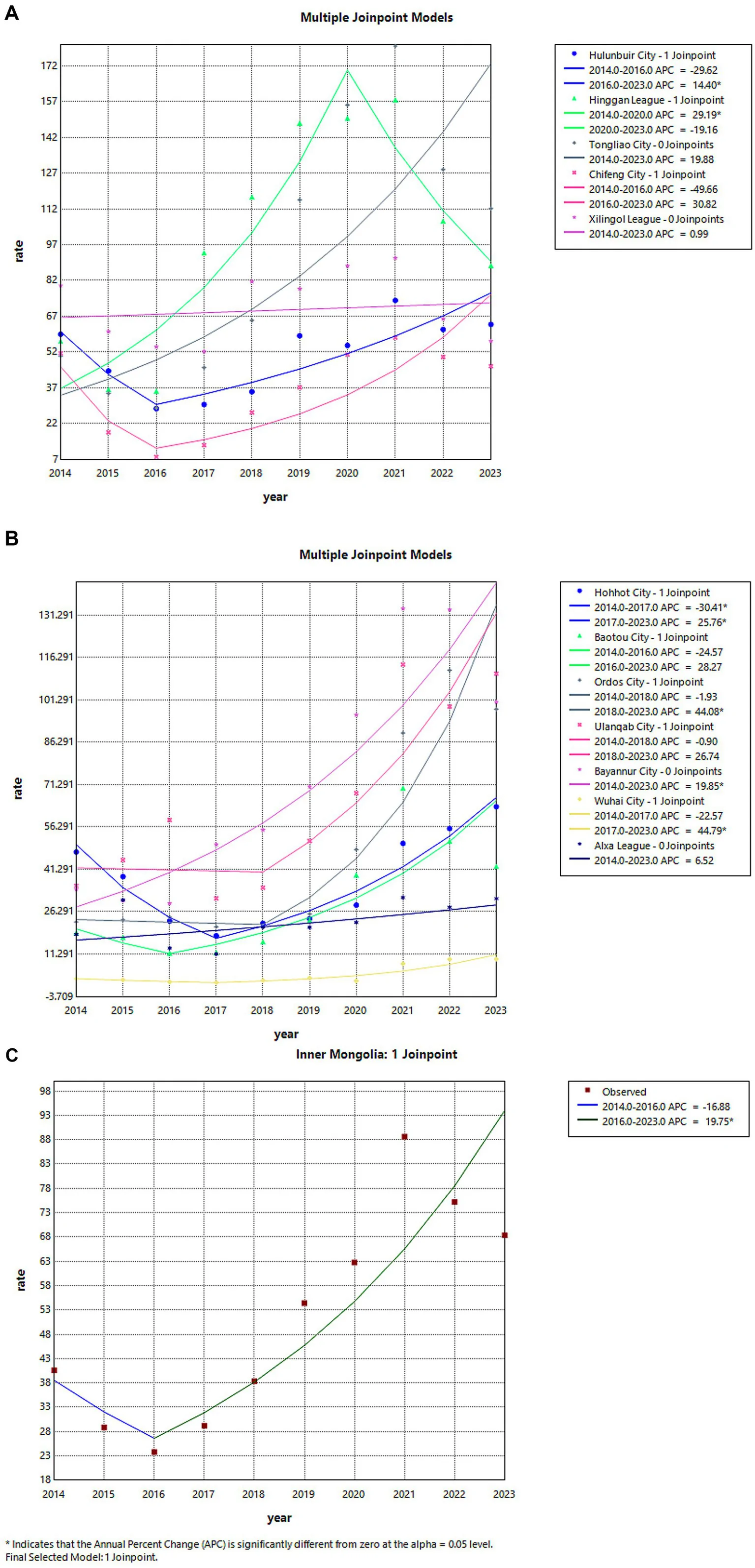
Joinpoint regression analysis in different regions of Inner Mongolia Autonomous Region, 2014–2023. **(A)** Eastern Inner Mongolia Region. **(B)** Western Inner Mongolia Region. **(C)** Whole Inner Mongolia.

### 3.2 Spatial distribution patterns

From 2014 to 2023, the spatial distribution of human brucellosis incidence in the Inner Mongolia Autonomous Region exhibited distinct dynamic evolution characteristics. In 2014, high-incidence areas were concentrated in the northern region, while only scattered secondary high-incidence zones existed in the western and central-eastern regions. From 2015 to 2017, these high-value areas in the north gradually subsided; the region was predominantly characterized by low-incidence areas, reflecting a pattern of low-level sporadic transmission. Starting in 2018, the epidemic entered a resurgence period, with the extent of secondary high-incidence areas expanding progressively from west to east and from south to north. Between 2019 and 2023, high incidence rates persisted, forming a stable core high-incidence belt across the traditional pastoral areas of the central-eastern and southern regions. Multiple high-value clusters emerged in the southern and southwestern parts of the province. With the central-eastern and southern regions serving as the core high-incidence zones, the epidemic reflected a region-wide prevalence (Figure 3).

**Figure 3:**
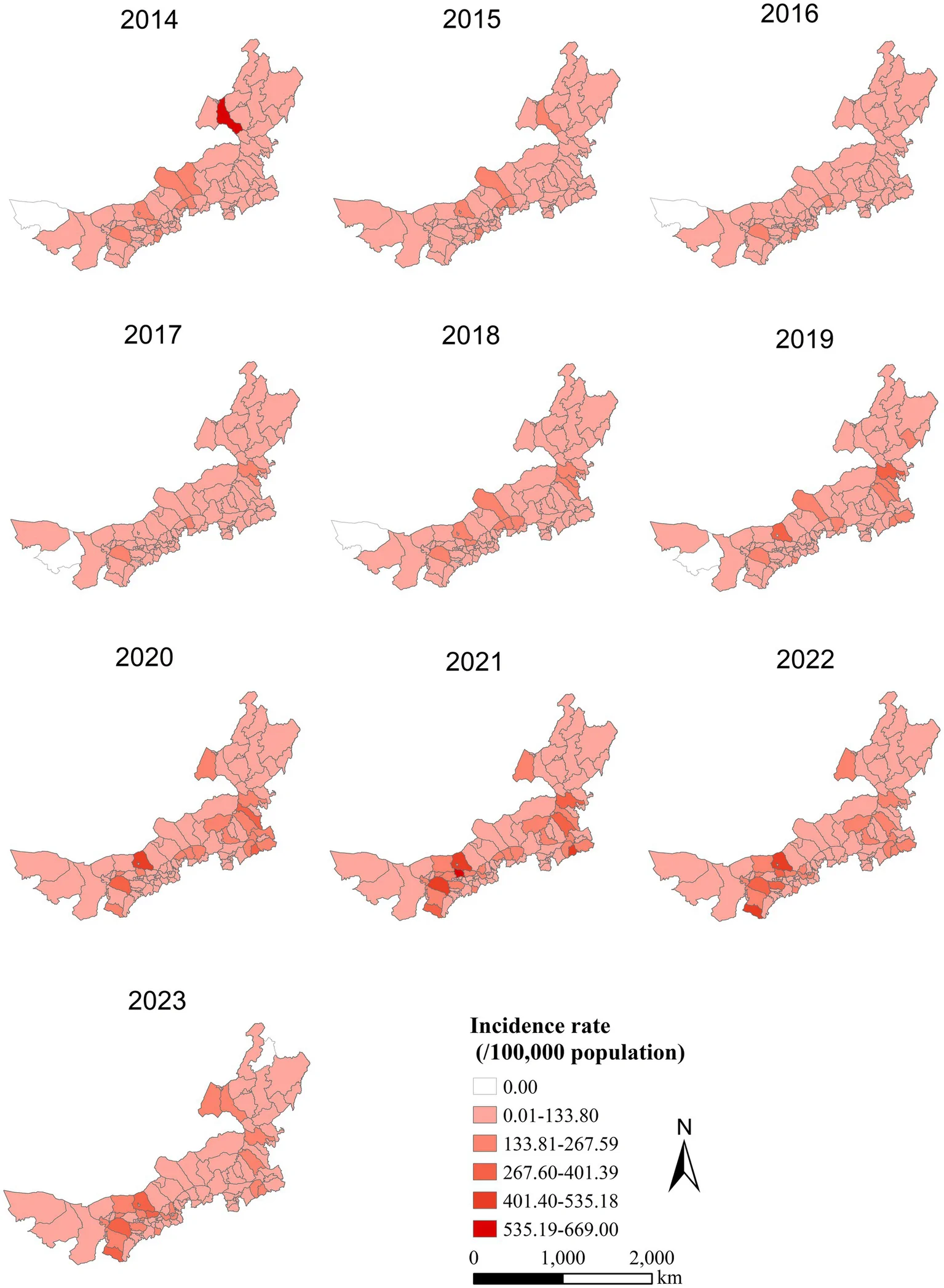
Spatial distribution of annual human brucellosis incidence in Inner Mongolia Autonomous Region.

Results from the spatial autocorrelation analysis indicated a global Moran’s *I* of 0.595 for the Inner Mongolia Autonomous Region from 2014 to 2023 (*p* < 0.001), suggesting a significant and positive spatial correlation throughout the entire study period. Except for 2014 and 2023, the Moran’s *I* values from 2015 to 2022 were all greater than 0 and statistically significant (*p* < 0.05), indicating that significant spatial correlation persisted across most years of the study (Table 3).

**Table 3:** Moran’s I index of brucellosis in Inner Mongolia, 2014–2023.

Year	Moran’s I	Z-score	*p*-values
2014	0.02	0.55	0.59
2015	0.16	2.53	0.01*
2016	0.27	4.20	<0.001*
2017	0.27	4.17	<0.001*
2018	0.27	4.25	<0.001*
2019	0.22	3.57	<0.001*
2020	0.16	2.64	0.01*
2021	0.12	2.02	0.04*
2022	0.12	2.02	0.04*
2023	0.11	1.77	0.08

Based on the spatio-temporal scan analysis of the 103 counties/districts in Inner Mongolia from 2014 to 2023, a total of 82 spatio-temporal clusters of brucellosis were identified. The primary cluster encompassed 20 counties/districts, accounting for 19.4% of the region’s total. It was primarily concentrated across the entirety of the Hinggan League (6/6) and Tongliao City (8/8), forming a continuous high-risk corridor. The most likely cluster (MLC) was located in central Hinggan League (RR = 5.99, *p* < 0.001), spanning from March 2019 to November 2023. Secondary clusters involved a total of 69 counties/districts, representing 67.0% of the 103 counties in the region. These areas were widely distributed across all 12 leagues and cities. Over time, the number of primary clusters gradually increased and their extent expanded, spreading from the central region of Inner Mongolia toward the eastern and western parts, while the number of secondary clusters showed a declining annual trend (Table 4; Figure 4).

**Table 4:** Spatiotemporal clusters of human brucellosis in Inner Mongolia, 2014–2023.

Scan time frame (year)	Cluster time(mm/dd-mm/dd)	Centroid (latitude, longitude)/ radius (km)	Cluster counties (n)	LLR	RR	*p*-values
2014	1/1–12/31	(40.38 N, 111.81E)/0	2	114.99	2.22	<0.001
2015	4/1–6/30	(48.22 N, 118.26E)/150.10	4	126.84	4.64	<0.001
2016	2/1–12/31	(39.83 N, 108.73E)/0	18	3020.03	2.40	<0.001
2017	5/1–11/30	(45.38 N, 121.59E)/82.35	3	1497.98	3.65	<0.001
2018	2/1–11/30	(41.09 N, 108.26E)/59.18	3	237.03	1.73	<0.001
2019	1/1–8/31	(45.38 N, 121.59E)/336.29	20	12025.73	2.32	<0.001
2020	12/1–12/31	(41.08 N, 107.07E)/137.98	7	34.5	2.08	<0.001
2021	2/1–8/31	(38.18 N, 107.47E)/331.19	18	3020.03	2.40	<0.001
2022	10/1–11/30	(38.18 N, 107.47E)/109.70	2	26.65	3.33	<0.001
2023	11/1–11/30	(43.62 N, 122.25E)/178.03	9	24.99	1.63	<0.001

LLR, Log likelihood ratio; RR, Relative risk.

**Figure 4:**
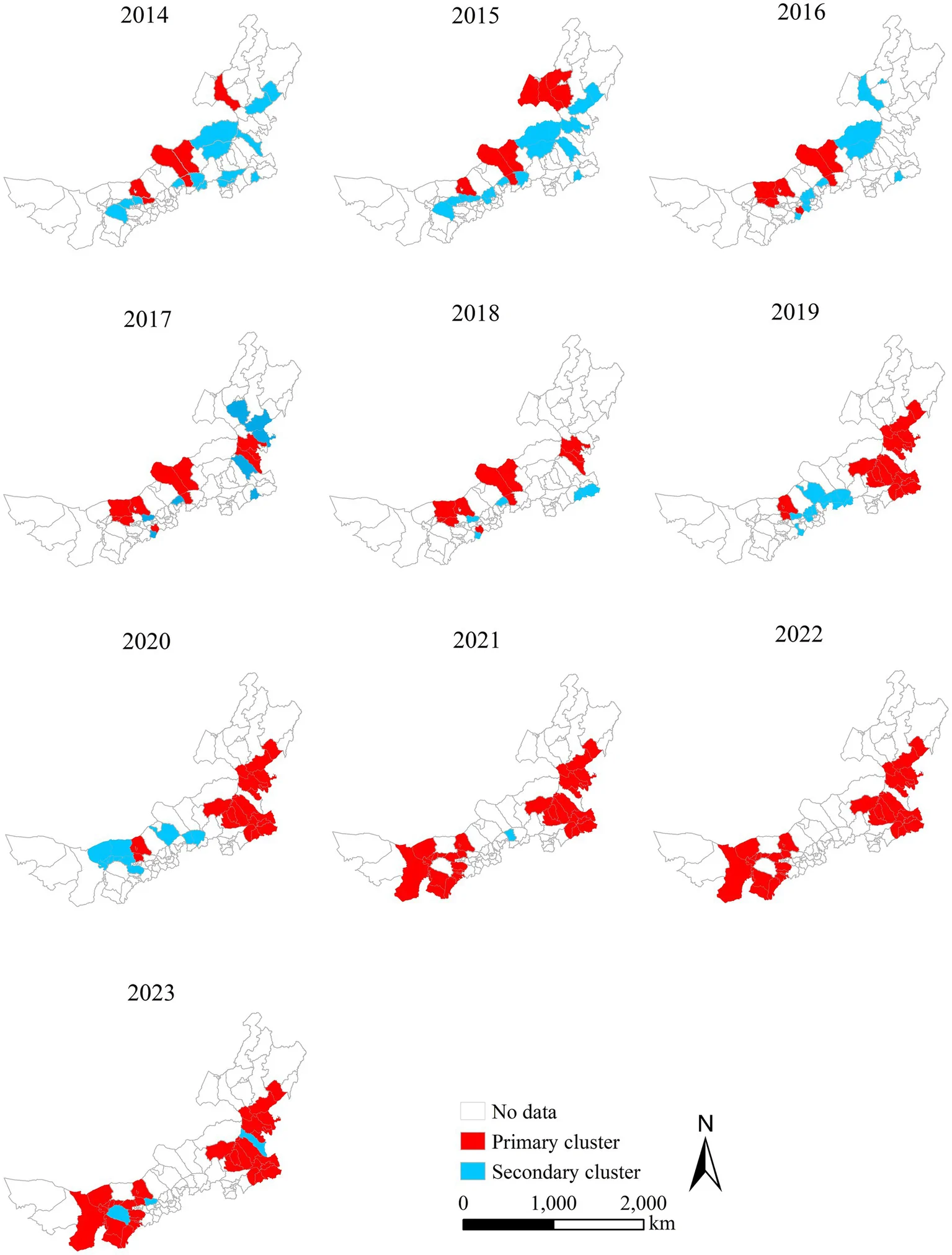
Spatial clustering of reported brucellosis cases in Inner Mongolia Autonomous Region, 2014–2023.

### 3.3 Disease burden analysis

From 2014 to 2023, the total *YLDs* attributable to brucellosis in Inner Mongolia reached 4033.61 person-years. The annual *YLD* values for the decade were 326.10 person-years in 2014, 240.07 person-years in 2015, 197.43 person-years in 2016, 237.63 person-years in 2017, 312.47 person-years in 2018, 441.57 person-years in 2019, 511.79 person-years in 2020, 673.16 person-years in 2021, 574.82 person-years in 2022, and 518.57 person-years in 2023. Temporal analysis revealed a fluctuating trend in annual *YLD* losses throughout the study period. Specifically, 2021 witnessed the peak *YLD* burden, contributing 16.69% of the total *YLDs*, whereas 2016 represented the lowest burden year, accounting for only 4.90% of the total.

High brucellosis disease burdens were concentrated in five prefecture-level leagues and cities, including Tongliao, Chifeng, Hinggan, Hulunbuir, and Ulanqab, with cumulative *YLDs* of 835.63, 469.53, 465.46, 375.91, and 370.21 person-years, respectively. Over the 10-year study period, the total indirect economic loss attributable to brucellosis in Inner Mongolia totalled 204.88 million CNY. The annual indirect economic losses from 2014 to 2023 were 15.45, 11.16, 9.06, 9.86, 13.93, 19.51, 23.63, 36.30, 34.13, and 31.84 million CNY, respectively.

Consistent with the *YLD* trend, the indirect economic loss exhibited an initial decline followed by a sustained rising trajectory across the study decade, with the annual maximum recorded in 2021 (constituting 17.71% of the total economic loss) and the minimum in 2016 (accounting for 4.42%). Stratified analysis revealed that the age-specific distribution of the indirect economic burden closely mirrored the patterns observed in *DALYs* (Figures 5, 6), highlighting that the disease and economic brunt was overwhelmingly concentrated in the active workforce. Except for 2014, when the 15–44 age group experienced a marginally higher burden (*DALY*: 144.29 person-years), the 45–59 age cohort consistently incurred the highest *DALYs* and indirect economic losses throughout the remainder of the study period. For this heavily impacted 45–59 cohort, both metrics peaked concurrently in 2021, with *DALYs* reaching a maximum of 307.94 person-years and indirect economic losses surpassing 20 million CNY. In contrast, the non-working-age populations faced a substantially lower burden. The group aged >60 years maintained intermediate and relatively stable losses, while the 0–14 years age group sustained the lowest impact across all cohorts. This pediatric group recorded minimal annual *DALYs* (remaining below 9.00 person-years) and a cumulative indirect economic loss of merely 0.65 million CNY, representing only 0.31% of the overall regional indirect economic burden of brucellosis.

**Figure 5:**
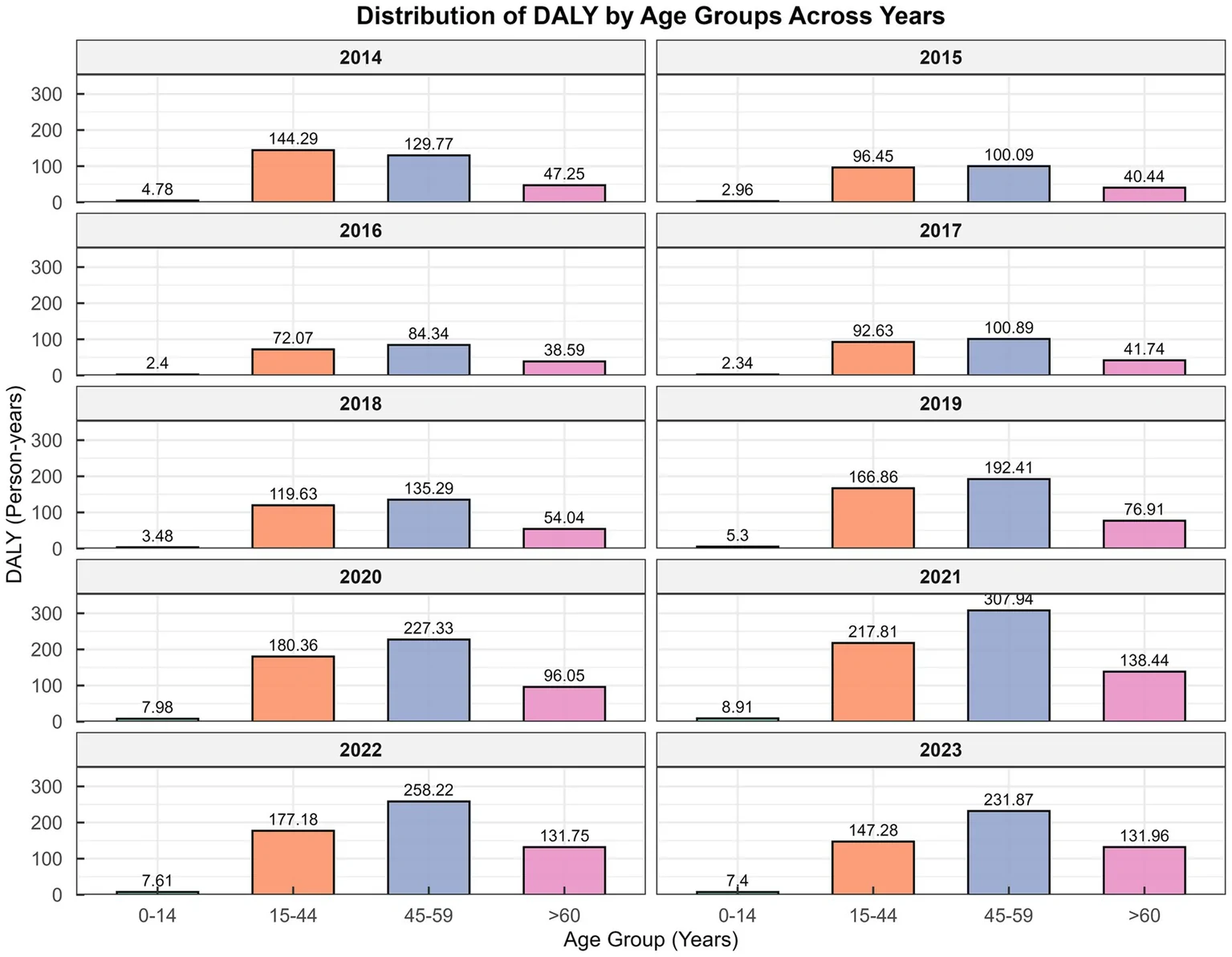
Distribution of *DALY* by age groups in Inner Mongolia Autonomous Region, 2014–2023.

**Figure 6:**
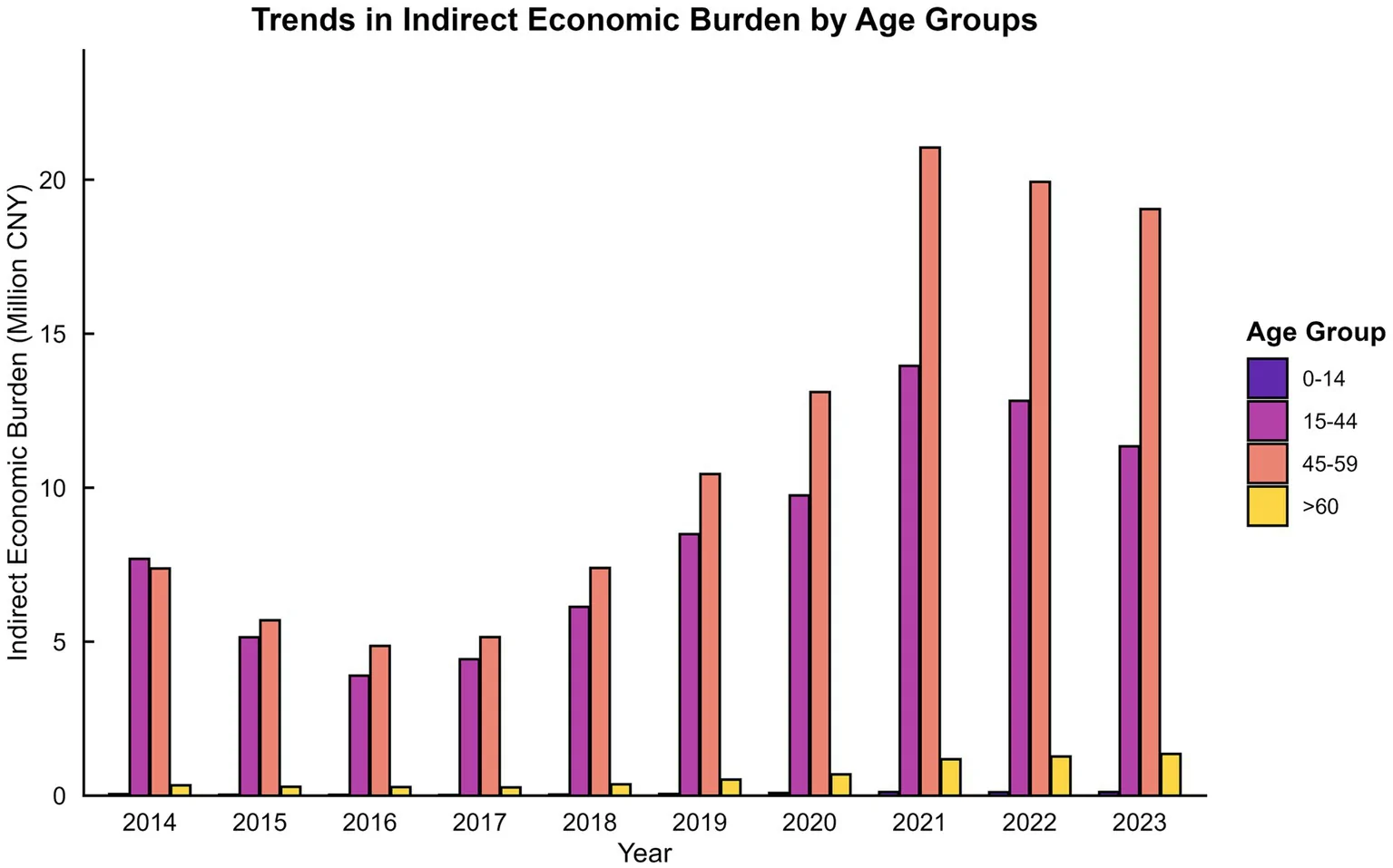
Trends in indirect economic burden by age groups in Inner Mongolia Autonomous Region, 2014–2023.

## 4 Discussion

This study performed a systematic epidemiological analysis of human brucellosis cases reported in the Inner Mongolia Autonomous Region between 2014 and 2023, elucidating the epidemiological characteristics and spatio-temporal distribution patterns of the disease over this decade. The incidence of brucellosis in this region demonstrated dynamic fluctuations superimposed upon an overall upward trajectory during the study period. Notably, the epidemiological features varied considerably across distinct epidemic stages, a progression consistent with the broader evolution of brucellosis epidemics documented in other provinces throughout China (3, 30, 31).

.Descriptive analysis revealed a pronounced demographic clustering pattern in the human brucellosis epidemic across Inner Mongolia. Farmers and herdsmen constituted the primary affected population, underscoring the occupational nature of brucellosis as a predominantly work-related zoonosis (32). The proportion of male patients was significantly higher than that of female patients, at approximately a 2:1 ratio, a finding consistent with global epidemiological trends reported for brucellosis (33). This sex-based disparity has been corroborated by numerous domestic and international studies. The observed difference is primarily attributed to the greater involvement of males in high-risk animal husbandry activities—including assisting in livestock parturition, slaughtering, and carcass processing—resulting in substantially elevated occupational exposure compared to their female counterparts (34). This skewed sex distribution is particularly pronounced in the pastoral areas of northern China, suggesting that targeted occupational protective interventions should prioritize the male farming and herding population.

Joinpoint regression analysis identified a single inflection point in the epidemiological trend of brucellosis in the Inner Mongolia Autonomous Region between 2014 and 2023. The incidence rate declined from 2014 to 2016, followed by a statistically significant increase from 2016 to 2023. Overall, the regional incidence demonstrated an upward trend over the study decade, consistent with findings reported in relevant domestic studies (24). The initial decline may be attributable to the joint human-animal brucellosis prevention and control program launched in 2010, which strengthened key interventions encompassing screening, quarantine, culling of infected livestock, immunization, and public health education. During this period, epidemic transmission was effectively contained. However, the subsequent upward trajectory after 2016 coincided with a marked rise in beef and mutton prices, which fundamentally shifted epidemiological dynamics. Rising commodity prices and growing consumer demand drove a substantial expansion of livestock production across Inner Mongolia, prompting increased public participation in animal husbandry, transportation, trade, and slaughtering activities. The strong correlation between the rapidly expanding livestock industry and the resurgence of brucellosis underscores the critical epidemiological interplay between animal reservoir hosts and human communities, as heightened direct contact with infected livestock correspondingly elevated human infection risk (35). In eastern Inner Mongolia, Hulunbuir, Chifeng, and the Hinggan League each exhibited one inflection point, characterized by alternating periods of decline and resurgence. No inflection points were detected in Tongliao and the Xilingol League, suggesting that the trends in these regions remained relatively stable or lacked distinct shifts in trajectory. The western region showed strong consistency in epidemiological trends. Except for the Alxa League, all other western divisions experienced a significant rise in incidence over the 10-year period (36). Notably, Hohhot, Baotou, and Ulanqab each exhibited clear inflection points, transitioning from initial phases of stability or minor fluctuation into periods of rapid growth. Collectively, these findings suggest that the western region warrants prioritization in future brucellosis prevention and control efforts. Regional authorities should refine integrated human-animal epidemic prevention strategies in alignment with current livestock industry development trajectories to effectively mitigate the risk of further escalating incidence (37).

In addition to spatial clustering, brucellosis cases displayed a marked seasonal pattern, with over half of all cases (55.3%) concentrated between March and July, peaking in May. As the surveillance data used here are aggregated at the monthly level, this temporal pattern should be interpreted as an ecological association rather than direct evidence of causation.

The incidence of human brucellosis in Inner Mongolia exhibited a significant positive spatial correlation, indicating clear spillover effects and transmission linkages driven by intensive regional livestock trade and the frequent movement of infected animals. Spatio-temporal scan analysis identified 82 clusters, with the primary core cluster encompassing Hinggan League and Tongliao. This prominent high-risk corridor is likely attributable to the high density of sheep farming and the region’s role as a critical logistics hub for livestock circulation between Inner Mongolia and Northeast China, which facilitates the cross-regional transmission of *Brucella* spp. The clustering period (2019–2023) highlights a persistent high-risk environment facilitated by traditional husbandry practices and frequent human-livestock contact during the spring lambing season identified above, where exposure to infected birth products has been reported as a primary transmission route (13). While core clusters expanded toward the east and west, reflecting dynamic shifts in grazing boundaries and market-driven animal transfers, secondary risk areas in western cities such as Bayannur and Ordos contracted annually (38). This contraction may be associated with the strengthening of veterinary control measures, including test-and-slaughter practices and mass vaccination campaigns, alongside a broader transition from fragmented backyard farming toward standardized intensive breeding; however, given the absence of longitudinal policy implementation data in the present dataset, this explanation remains preliminary and warrants further verification (39, 40). These findings underscore that the spatial persistence of brucellosis is deeply rooted in the socio-economic structure of major pastoral production areas, where livestock density, trade networks, and regional veterinary management efficacy dictate the epidemic’s trajectory (41). The vast distribution of pastures in Inner Mongolia is highly conducive to animal husbandry development, which in turn has exacerbated the prevalence of brucellosis. Previous studies have indicated that while “high-high” spatial clusters in Inner Mongolia are gradually decreasing, there is an observable spatial shift from the eastern to the western regions (42, 43). The epidemic intensity of brucellosis is highly correlated with the level of regional agricultural and pastoral development. The confluence of factors such as the rapid expansion of the breeding industry and the lack of self-protection awareness among herdsmen has driven the risk within core epidemic areas continuously upward, leading to its persistent outward diffusion.

This study found that human brucellosis imposed a sustained health and economic burden in Inner Mongolia from 2014 to 2023. The annual burden variations aligned with local epidemic trends, with both the disease burden and economic losses peaking in 2021. Significant regional disparities in disease burden were observed across leagues and cities. Traditional major pastoral areas such as Tongliao and Chifeng bore heavier burdens, which is directly linked to local breeding models and high population exposure risks. The working-age population accounted for the vast majority of indirect economic losses, whereas the proportional loss among children aged 0–14 was minimal. This corresponds with the demographic characteristics of brucellosis incidence and the differential productivity weights across age groups (12, 44). However, it is crucial to acknowledge that our calculations may underestimate the actual disease burden. By utilizing a fixed disability weight of 0.15 and an average disease duration of 75 days based on WHO parameters, this study predominantly reflects the acute phase of the infection. In clinical reality, brucellosis manifests in acute, subacute, and chronic stages; patients progressing to the chronic stage frequently endure significantly prolonged illness durations and higher disability weights due to severe, long-term complications. Despite this conservative estimation, these findings elucidate the priority regions and high-risk populations for brucellosis prevention and control in Inner Mongolia, providing a reference for the targeted implementation of local interventions (45).

Several limitations of the present study warrant acknowledgement. First, as this study exclusively quantified the indirect economic burden of brucellosis, future research should incorporate direct medical costs, including hospitalization expenditures, diagnostic costs, and treatment expenses, to enable a more comprehensive assessment of the total disease burden. Second, the absence of meteorological variables and the lack of adjustment for regional disparities in healthcare resource availability and service capacity constrain a thorough understanding of the multifactorial determinants underlying the observed spatio-temporal clustering patterns. Third, the *YLD* calculation in this study applied a fixed disability weight of 0.15 and a uniform disease duration of 75 days across all cases, based on WHO recommended parameters for acute brucellosis. This approach, while methodologically consistent with prior disease burden studies, predominantly captures the acute phase of the disease and likely underestimates the true burden attributable to chronic sequelae, such as recurrent arthralgia, spondylitis, and fatigue, which can persist for months to years in a subset of patients. Owing to the unavailability of clinical follow up data distinguishing acute from chronic disease courses in the current surveillance dataset, a differentiated disability weighting scheme could not be applied. Future studies incorporating clinical staging data are needed to conduct sensitivity analyses using stratified disability weights for acute and chronic brucellosis, which would yield a more accurate and comprehensive estimate of the overall disease burden.

## 5 Conclusion

Human brucellosis in Inner Mongolia remains a significant public health challenge, characterized by pronounced spatio-temporal clustering and a persistent trend of spatial diffusion. Timely and effective prevention and control measures tailored to high-risk areas and vulnerable populations are therefore urgently warranted, with the overarching goal of reducing brucellosis transmission and safeguarding public health. Future research should integrate individual-level epidemiological data and systematically investigate the potential impacts of climate change on long-term disease trends, thereby providing a more robust scientific foundation for the phased control and long-term mitigation of brucellosis burden in the region.

## Data Availability

The data that support the findings of this study are available on request from the corresponding author. The data are not publicly available due to privacy restrictions.
